# General practitioners’ perceptions of population based bowel screening and their influence on practice: a qualitative study

**DOI:** 10.1186/s12875-017-0610-8

**Published:** 2017-03-15

**Authors:** Greer Dawson, Melanie Crane, Claudine Lyons, Anna Burnham, Tara Bowman, Donna Perez, Joanne Travaglia

**Affiliations:** 1 Sax Institute, Level 13, Building 10, 235 Jones Street, Ultimo, NSW Australia 2007; 2 School of Public Health and Community Medicine, Samuels Building, University of New South Wales, Sydney, NSW Australia 2052; 3 Prevention Research Collaboration, The Charles Perkins Centre, Level 6, The Hub, School of Public Health, University of Sydney, Sydney, NSW Australia 2006; 4 NSW Department of Premier and Cabinet, 52 Martin Place, Sydney, NSW Australia 2000; 5 Cancer Institute NSW, Australian Technology Park, Level 9, 8 Central Avenue, Sydney, NSW Australia 2015; 6 Faculty of Health, University of Technology Sydney, Level 7, 235 Jones Street, Sydney, NSW Australia 2007

**Keywords:** Bowel cancer, Bowel screening, FOBT, General practitioners, Qualitative, Cancer prevention, Early detection

## Abstract

**Background:**

Although largely preventable, Australia has one of the highest rates of bowel cancer in the world. General Practitioners (GPs) have an important role to play in prevention and early detection of bowel cancer, however in Australia this is yet to be optimised and participation remains low. This study sought to understand how GPs’ perceptions of bowel screening influence their attitudes to, and promotion of the faecal occult blood test (FOBT), to identify opportunities to enhance their role.

**Methods:**

Interviews were conducted with 31 GPs from metropolitan and regional New South Wales (NSW), Australia. Discussions canvassed GPs’ perceptions of their role in bowel screening and the national screening program; perceptions of screening tests; practices regarding discussing screening with patients; and views on opportunities to enhance their role. Transcripts were coded using Nvivo and thematically analysed.

**Results:**

The study revealed GPs’ perceptions of screening did not always align with broader public health definitions of ‘population screening’. While many GPs reportedly understood the purpose of population screening, notions of the role of asymptomatic screening for bowel cancer prevention were more limited. Descriptions of screening centred on two major uses: the use of a screening ‘process’ to identify individual patients at higher risk; and the use of screening ‘tools’, including the FOBT, to aid diagnosis. While the FOBT was perceived as useful for identifying patients requiring follow up, GPs expressed concerns about its reliability. Colonoscopy by comparison, was considered by many as the gold standard for both screening and diagnosis. This perception reflects a conceptualisation of the screening process and associated tools as an individualised method for risk assessment and diagnosis, rather than a public health strategy for prevention of bowel cancer.

**Conclusion:**

The results show that GPs’ perceptions of screening do not always align with broader public health definitions of ‘population screening’. Furthermore, the way GPs understood screening was shown to impact their clinical practice, influencing their preferences for, and use of ‘screening’ tools such as FOBT. The findings suggest emphasising the preventative opportunity of FOBT screening would be beneficial, as would formally engaging GPs in the promotion of bowel screening.

## Background

Although largely preventable, Australia has one of the highest rates of bowel cancer in the world [1]. Early diagnosis increases the likelihood of successful treatment, however fewer than 40% of bowel cancers are detected early [2]. A Faecal Occult Blood Test (FOBT) increases early detection of bowel cancer [3–6], while detection and removal of pre-cancerous lesions reduces overall incidence [7]. In Australia, *The Clinical Practice Guidelines for the Prevention, Early Detection and Management of Colorectal Cancer* (2005) recommend biennial FOBT screening in the average-risk population [8]. Population screening aims to reduce the burden of disease in the general population by increasing the likelihood of identifying individuals with the disease before symptoms develop [9]. It differs significantly from detecting symptomatic cases or ‘individual case finding’ in asymptomatic patients with certain risk factors [10].

The National Bowel Cancer Screening Program (NBCSP) commenced in Australia in 2006. The program uses an immunochemical FOBT and operates by mailing invitations and FOBT kits directly to eligible individuals’ home addresses based on Medicare data [11]. Currently, FOBT screening is offered on a 5-yearly basis commencing from age 50, with progression to biennial screening by 2020 underway [11]. This differs from the bowel screening program in the United Kingdom, which offers biennial FOBT screening from age 60 [12], or Japan which offers annual screening from age 40 . The United States has no organised program, however screening with FOBT, sigmoidoscopy or colonoscopy (depending on individual risk factors) is promoted through guideline dissemination and media campaigns.

The success of population screening programs depends on high participation, yet suboptimal participation in bowel screening is common around the world [13]. In Australia, participation in the NBCSP is around 30% [11], although it should be noted that some individuals report screening outside of the NBCSP [14] and are therefore not captured in this figure. Poor participation may be attributable to a range of factors including poor knowledge and awareness [15–17], confusion regarding recommendations and eligible ages which have changed over time [15], and lack of General Practitioner (GP) endorsement [17]. There is however, potential to reduce bowel cancer incidence through a concerted approach to screening.

In Australia, GPs are the first point of contact that individuals have with the health system. On average, Australians visit their GP around five times per year [18]. At present, GPs have no formal role in recruiting individuals into the NBCSP however have considerable potential to encourage participation. GPs play a significant role in promoting breast and cervical screening in line with current recommendations and acknowledge this as part of their approach to preventative health [19]. In relation to bowel screening, research suggests GP endorsement substantially increases compliance [20–24], however in Australia there has not yet been a coordinated effort to optimise the role of GPs in promoting bowel-screening participation.

The importance of screening is gaining attention in primary care, with national calls for health promotion, prevention and screening to be recognised as core roles in general practice [25]. GPs also report feeling positive towards undertaking prevention and health promotion activities, however increasing expectations of GPs to undertake a range of preventative activities in addition to diagnosing and treating patients should be acknowledged. Discussions about screening can be inhibited by factors such as time, distractions during consultations, multiple health concerns, and uncertainty about particular screening recommendations [26]. Time pressures and high workload are cited as frequent barriers [19, 27–29]. GPs’ attitudes and level of confidence to initiate health promotion interventions also influence the inclusion of such activities in their routine practice [27, 30].

Existing research suggests the success of population-based screening will largely be determined by GP attitudes and support of the FOBT [31, 32]. It has also been shown that some GPs have concerns about the efficacy of the FOBT [33], however it remains unclear as to how this fits with their understanding of population based screening and prevention, and to what extent this shapes their clinical practice. To date we have been unable to locate any studies that examine specifically how GPs’ perceive population based bowel screening in relation to cancer prevention, and how this affects their use of the FOBT. To address this gap, we sought to understand how GPs’ perceptions of bowel screening influence their attitudes to, and promotion of the FOBT.

## Methods

This study was conducted in 2014, eight years after the commencement of the NBCSP. A qualitative approach was selected to enable a nuanced understanding of GPs perceptions and attitudes. Semi-structured interviews were conducted with 31 GPs from around NSW who were recruited through research panels. Research panels are groups of pre-screened respondents who have expressed a willingness to participate in research. Purposive sampling was used to ensure representation of GPs from a variety of metropolitan (*n* = 16) and regional (*n* = 15) locations in NSW. Proportions of males and females were approximately equal. The sample was structured to include GPs with a higher proportion of patients of eligible screening age (50 years and over). This was done via a screening question asked in the recruitment phase that excluded GPs who indicated they had a relatively low proportion of patients aged over 50.

Interviews were conducted once, either over the phone or in-person after hours in the clinic, and were approximately 30 min in duration. Two experienced independent interviewers from a specialised qualitative research firm were engaged to conduct the interviews. All members of the research team, including the external interviewers, were independent of the primary care sector and no prior relationship existed between the participants and the researchers/interviewers. Participants were not informed of the research topic in advance and were reimbursed for their time. The interviews followed a discussion guide developed by the authors (Table 1) and pilot tested by the interviewers. No new themes emerged following the last few interviews indicating thematic saturation was reached.

**Table 1:** Discussion guide

1. What do you see as your role as a GP in cancer screening (general)?
2. What do you see as your role as a GP in *bowel* screening?
3. What factors influence whether or not you discuss bowel screening with patients? (*Prompt for barriers and enablers*)
4. What advice do you give patients regarding bowel screening, and does this differ depending on the patient? Can you please elaborate?
5. What is your view of different bowel cancer screening tests (FOBT, colonoscopy, flexible sigmoidoscopy)?
6. From your perspective, how willing are patients to undertake bowel screening? What factors do you think affect this (patient willingness)?
7. What sources of information about bowel cancer / bowel cancer screening do you use?
8. What bowel cancer screening guidelines do you know about? What is available? How useful?
9. What do you know about the NBCSP?
10. What is your opinion of the NBCSP / Understanding of screening eligibility / The role of GPs in the program?
11. What is your experience of the NBCSP in your practice?
12. When you feel that a patient should have an FOBT, what do you do?
13. What do you do with patients not eligible for the NBCSP?
14. What could help enhance the role of primary care in bowel screening to improve screening and early detection?

Interviews were transcribed verbatim and coded using NVivo by the first author, GD. A thematic analysis was developed around the research objectives and emergent trends in the data using the approach developed by Braun and Clarke (2006) [34]. Rigour was addressed through an iterative process of constant comparison and the analysis process was documented to ensure process auditability [35]. A second investigator (JT) reviewed a random sample of transcripts and analysis to check the interpretation then the final analysis was developed via a consultative process involving all authors. Reflexivity was facilitated through an iterative process of analysis, with assumptions underpinning interpretations exposed through comparisons between authors’ interpretations and with existing evidence. Differences were resolved through discussion [36]. Participants were not recontacted or provided with copies of the transcripts for verification, however the authors crosschecked the final thematic analysis with the interviewers who had conducted a separate independent analysis.

Ethics approval was granted by the University of New South Wales Human Research Ethics Advisory Panel.

## Results

The results are presented in two sections. The first examines GPs’ perceptions of screening, while the second section explores their preference for and use of different screening tools. The range of perspectives and opinions are illustrated in each section. Overall, GPs acknowledged they had a potentially important role to play in bowel screening, and support for the NBCSP was widespread despite confusion about its delivery. GPs articulated a range of interpretations of what their role might entail, including providing education, encouraging compliance, detecting patients who had been missed by the NBCSP, and facilitating alternative screening pathways. A few GPs observed that the existence of the NBCSP meant their role was limited. Although GPs saw their role in screening to be important, there was variation in how this translated in their everyday practice, with perceptions of screening observed to exert an important influence on both attitudes and practice.

### Perceptions of ‘screening’

GPs’ perceptions of bowel screening were varied. A few GPs had a detailed understanding of the meaning of population screening and the importance of targeting the asymptomatic population to enable prevention and early detection of bowel cancer. The majority, however, referred to screening in terms of identifying risk factors and facilitating screening for those assessed to be at higher risk. GPs also referred to screening as part of the diagnostic process.

#### The screening ‘process’

Most GPs discussed screening in terms of a diagnostic or risk assessment ‘process’. GPs spoke about their role in identifying patients who *should* be screened due to pre-existing risk factors. Assessing whether an individual required screening was seen as the first step in the ‘screening’ process.

In accordance with the National Guidelines [8], age was a key factor in determining whether screening was a priority. Other risk factors GPs considered included, whether patients had a first-degree family history of bowel cancer, and the presence or history of gastrointestinal symptoms.

*‘My role is basically to find the right kind of patient to screen, so either age related or [if] they have a strong family history…’*

Male, regional

Screening was also referred to as something that should be done to address symptoms. This GP was asked if she had a system for raising screening with patients. She responded:

*‘You ask them if they have any gastrointestinal symptoms and sure, if there was some suspicion, then I’d probably put an occult blood on the form’*

Female, regional

Some GPs talked about bowel screening specifically in terms of aiding diagnosis. In these cases ‘screening’ was used to exclude certain conditions, including cancer, and hence formed part of the process of diagnosing rather than screening per se.

*‘There are acute triggers, like patients who present with painful motions, an unexplained change in bowel habits or new cases of anaemia, that lead me to recommend FOBT, not so much because I suspect cancer but more because I want to rule it out as a possible cause of the patient’s symptoms.’*

Male, regional

#### Screening for early detection and prevention

While the majority of GPs described screening in terms of an assessment and diagnosis process, a few spoke of its role in early detection and prevention.

*‘I think the studies so far have shown that if we do screen the population at certain ages, that it does seem to pick up some of the bowel cancers earlier and therefore it’s a good thing to do.’*

Male, metropolitan

*‘Look, we talk about prevention really. It’s good to practice preventative medicine. So pap smears, mammograms, for cervical cancer and breast cancer … and faecal occult blood for bowel cancer.’*

Female, metropolitan

The role of population screening and the importance of targeting the asymptomatic population was also raised by some GPs.

*‘What I see is to pick up cancer in the asymptomatic population, and the higher the pick-up rate, the better the outcome because it’s fixable in the early stages, it’s treatable. So my role is to pick up [cancer] early, as soon as possible.’*

Female, metropolitan

#### Use of screening tools

GPs perceptions of three types of bowel screening tools were explored, the colonoscopy, the FOBT and the flexible sigmoidoscopy, although the latter was reported as being used infrequently. GPs’ attitudes and stated preferences tended to reflect their conceptualisation of screening and the perceived utility of specific methods as diagnostic tools.

For GPs who conceptualised screening in terms of identifying and assessing patients at higher risk (patients with a family history or symptoms), then, in accordance with the National Guidelines [8], colonoscopy was overwhelmingly the preferred methodology.

*‘We have to encourage a vigilant approach, especially in those with a change in bowel habits or close family history of bowel cancer, or for patients who have had polyps removed in the past … These people need colonoscopy and I refer them to a gastroenterologist to check for bowel cancer.’*

Male, regional

GPs who viewed screening in terms of its diagnostic end point (detection of cancer) also tended to perceive colonoscopy was the gold standard ‘screening’ tool as it enabled a definitive diagnosis to be made.

*‘Well, I think at the moment, that [colonscopy’s] the best way or the gold standard to check for any bowel disease – bowel polyps and bowel cancer’*

Male, metropolitan

*‘I mean, a screening test – it has to be valuable and diagnostic at the same time.’*

Female, metropolitan

By the same token, owing to its limitations as a diagnostic test, the FOBT was perceived by some to be inferior.

*‘A faecal occult blood test is a very basic screening tool which does not necessarily pick up bowel cancer. … I would only recommend it for the person who is not agreeable to go for a colonoscopy.’*

Female, metropolitan

Several GPs questioned the accuracy of the FOBT, expressing concerns about the number of false positives.

*‘You only need a small amount of blood … and it will come up positive in your stool, so I usually send them directly for a colonoscopy.’*

Female, regional

Despite both its limitations as a diagnostic tool, and concerns regarding accuracy, many GPs still saw the FOBT as a useful tool that could indicate where further investigation was warranted.

*‘It’s a good rough tool. Of course there’s a significant rate of false positives, and no doubt a rate of false negatives as well, but it’s not an unreasonable rough tool’*

Male, regional

GPs tended to have more positive perceptions of the FOBT where their concept of screening was population based. For these GPs, FOBT was recognised as an economical, non-invasive tool for identifying individuals requiring further clinical investigation.

*‘It seems that there’s really only one screening test: faecal occult blood testing, FOBT … FOBT is the best population-screening tool just because it is cheap and zero-risk to the patient. Of course, it is not a perfect diagnostic test but it does help identify people for further investigation …’*

Female, metropolitan

## Discussion

Increasing FOBT screening as a population measure is important given its potential to facilitate prevention and early detection of bowel cancer and reduce overall disease incidence. GPs have a key role to play in encouraging screening participation [20–23], therefore strategies to formally engage them in the promotion of routine asymptomatic screening to patients should be considered [37]. The present study provides important insight into how GPs both conceptualise screening and promote use of the FOBT in their clinical practice, pointing to both opportunities and strategies to enhance their role.

This study builds on previous research that has identified the importance of GPs’ knowledge and attitudes in shaping their clinical practice in relation to bowel screening [38, 39] by exploring *how* these factors impact on their practice. A novel finding derived from this research shows that GPs’ concepts of screening did not always align with broader public health definitions of ‘population screening’ [9]. In particular, many GPs did not appear to have a well-formed understanding of the role of asymptomatic bowel screening in detecting pre-cancerous lesions and thus the role of screening in bowel cancer prevention. GPs frequently construed screening as either part of a diagnostic process, or reserved for patients with individualised risk factors or symptoms. These findings suggest GPs interpret ‘screening’ within their localised clinical context, rather than within the broader perspective of population level prevention. Conceptualising screening as either assessing risk or aiding diagnosis signifies that screening was often understood as opportunistic detection or individual case finding. Given clinicians generally operate within the patient-centric Hippocratic tradition, where how an intervention helps an individual patient is the most important determinant, it makes sense that they are less likely to think in terms of ‘population’ benefits [40]. The diagnostic emphasis of the medical model amalgamated with the pressures of time and workload further compound this [29, 38].

Moreover, we found that GPs’ perceptions of screening influenced both their preferences for, and use of different screening tools, with the FOBT often mistakenly evaluated in terms of its diagnostic capabilities. For GPs who viewed screening as part of a diagnostic process, it seems logical that they might prefer a test such as colonoscopy, which can more effectively aid diagnosis. Similarly, for GPs who did not make a clear conceptual distinction between screening and diagnosis, the FOBT was often mistakenly evaluated in terms of its capabilities as a diagnostic test. As the FOBT is non-specific and thus limited in its diagnostic capabilities it follows that colonoscopy would be preferred. Concerns about false positives (where a cancer diagnosis does not follow), suggests that the FOBT seemed to be widely misunderstood as to its purpose. Such concerns amongst GPs have been reported previously by studies undertaken in the United States [41] and Australia [33]. FOBT is not a diagnostic test for bowel cancer; rather it is a test for blood in the stool for which it is very sensitive. Perceived in those terms, concerns about false positives should not be widespread. GPs tended to have a more positive view of the FOBT when it was perceived as a population-based screening tool to indicate where further investigation was required. Having a poor understanding of the role of screening in the prevention of disease is a barrier to the integration of routine asymptomatic bowel screening activities in general practice.

The findings from this study support existing research that has found the perceived efficacy of screening to be a determinant of clinical practice [32, 38]. To address these issues, we need strategies to engage and encourage GPs to adopt more preventive approaches within their current models of care. GPs need adequate information focused around the effectiveness of routine screening programs, including the proportion of false negatives and false positives, in order to effectively promote screening [42]. Targeted information around the natural history of bowel cancer including pre-cancerous lesions and the role of asymptomatic screening in prevention would also provide a useful framework from which to enhance GPs’ understandings of population screening [37]. Formally engaging GPs in the promotion of bowel screening would likely raise the profile of the disease and reinforce the importance of prevention and early detection. In France, GPs are directly involved in administering the FOBT screening program, which has been shown to have implications for increased participation, particularly when the GP delivers the test [38]. The involvement of both GPs and patients in a shared care approach also provides an opportunity to facilitate awareness and promote the value of screening [38].

Practice-based strategies that prompt GPs to discuss screening with eligible patients should also be implemented [43, 44]. This could include establishing systems that enable GPs to identify patients who are eligible for screening and trigger a reminder at the time of consultation. Other strategies such as organising special clinics that focus on preventative activities or involving practice nurses in screening activities may assist in addressing some of the practical barriers faced by GPs in promoting screening to patients [45]. Engaging patients while they are waiting for a GP visit by asking them to complete a bowel screening self-assessment, may be another useful way to initiate discussions about screening and has the added advantage of fostering a shared decision-making approach.

### Limitations

Although we sought diversity in our sample, it is possible that this may have been constrained by the recruitment of participants from research panels, which may limit the applicability of our findings to the broader population of GPs in NSW. Furthermore, self-report measures may not always reflect actual practice. Despite these limitations, the findings highlight important issues, which if adequately addressed may contribute to increasing bowel screening participation in NSW.

## Conclusion

The results of this study show that GPs’ interpretations of screening do not necessarily align with defined notions of population screening and the importance of FOBT screening may therefore be under realised. The findings illustrate how perceptions of screening influence attitudes and practice, and suggest a greater emphasis on the preventative opportunity of FOBT screening would be beneficial, as would formally engaging GPs in the promotion of bowel screening. This may assist in re-shaping how some GPs view the FOBT and utilise it in their clinical practice.

## Abbreviations

FOBT: Faecal occult blood test
GP: General Practitioner
NBCSP: National Bowel Cancer Screening Program.
